# Current Status of Epidemiology, Diagnosis, Therapeutics, and Vaccines for Novel Coronavirus Disease 2019 (COVID-19)

**DOI:** 10.4014/jmb.2003.03011

**Published:** 2020-03-21

**Authors:** Dae-Gyun Ahn, Hye-Jin Shin, Mi-Hwa Kim, Sunhee Lee, Hae-Soo Kim, Jinjong Myoung, Bum-Tae Kim, Seong-Jun Kim

**Affiliations:** 1 Center for Convergent Research of Emerging Virus Infection, Korea Research Institute of Chemical Technology, Daejeon 344, Republic of Korea; 2 Bioenvironmental Science and Toxicology Division, Gyeongnam Branch Institute, Korea Institute of Toxicology, Jinju 5834, Republic of Korea; 3 Korea Zoonosis Research Institute and Genetic Engineering Research Institute, Jeonbuk National University, Jeollabuk-do 54896, Republic of Korea

**Keywords:** 2019-nCoV, COVID-19, SARS-CoV-2, coronavirus, outbreak

## Abstract

Coronavirus disease 2019 (COVID-19), which causes serious respiratory illness such as pneumonia and lung failure, was first reported in Wuhan, the capital of Hubei, China. The etiological agent of COVID-19 has been confirmed as a novel coronavirus, now known as severe acute respiratory syndrome coronavirus 2 (SARS-CoV-2), which is most likely originated from zoonotic coronaviruses, like SARS-CoV, which emerged in 2002. Within a few months of the first report, SARS-CoV-2 had spread across China and worldwide, reaching a pandemic level. As COVID-19 has triggered enormous human casualties and serious economic loss posing global threat, an understanding of the ongoing situation and the development of strategies to contain the virus’s spread are urgently needed. Currently, various diagnostic kits to test for COVID-19 are available and several repurposing therapeutics for COVID-19 have shown to be clinically effective. In addition, global institutions and companies have begun to develop vaccines for the prevention of COVID-19. Here, we review the current status of epidemiology, diagnosis, treatment, and vaccine development for COVID-19.

## Introduction

In December 2019, cases of serious illness causing pneumonia and death were first reported in Wuhan, the capital of Hubei, China. Soon after, the number of cases soared dramatically, spreading across China and worldwide. As of March 25, more than 400,000 cases of the disease have been confirmed with over 18,000 deaths. The causative agent of the disease has been confirmed as a novel coronavirus (CoV). The World Health Organization (WHO) announced the official name of the disease as “coronavirus disease 2019 (COVID-19)” and now publicly refers to the virus as “the COVID-19 virus” (formerly known as “2019-nCoV”, or "Wuhan Coronavirus"). Analysis of the viral genome has revealed that the new coronaviruse is phylogenetically close to severe acute respiratory syndrome coronavirus (SARS-CoV) [1], the causative agent of a viral outbreak in 2002. Thus, the new coronavirus has been named “SARS-CoV-2” by the International Committee on Taxonomy of Viruses (ICTV) and other virologists [2].

Coronaviruses are enveloped, positive-sense single-stranded viruses ((+)ssRNA virus) belonging to the family *Coronaviridae*. Most coronaviruses have 8-10 open reading frames (ORFs). ORF1a and ORF1b are translated into polyprotein 1a (pp1a) and pp1ab, which are processed by viral proteases to produce 16 non-structural proteins containing RNA-dependent RNA polymerase enzyme (RdRp). The viral RNA is replicated through transcription of a minus-strand template by RdRp. During replication, coronaviruses generate 6-9 subgenomic mRNAs (sgmRNAs), which lead to translation of accessory and structural proteins from downstream ORFs [3]. Spike (S), envelope (**E**), membrane (M), and nucleocapsid (N) proteins, necessary for completion of a viral replication cycle, are translated from sgmRNAs [4].

Many coronaviruses are known to infect humans and various animals. In general, 15-30% of common colds are caused by human coronaviruses (HCoVs) including HCoV-229E, HCoV-NL63, HCoV-OC43, and HCoV-HKU1 [5]. However, some coronaviruses from animal reservoirs can be transmitted to humans causing outbreaks in the human population. The SARS-CoV outbreak in 2002 originated from bats in China [6] and the Middle East respiratory syndrome coronavirus (MERS-CoV) outbreak in 2012 from dromedary camels, though also likely transmitted from bats, in the Middle East [7]. Although the origin of the SARS-CoV-2 outbreak has not yet been identified, it has been reported that SARS-CoV-2 might be transmitted by bats [8], snakes [9], or pangolins [10, 11]. Unlike HCoVs, these zoonotic viruses infect both humans and various animals and cause severe respiratory illnesses such as acute respiratory distress syndrome (ARDS) and pneumonia, leading to death [12, 13]. The symptoms of COVID-19 are more severe in older age groups with comorbidities, while allergic diseases, asthma, and chronic obstructive pulmonary disease (COPD) are also risk factors [14, 15].

Since 2000, various zoonotic coronaviruses have been circulating in the animal reservoir [11, 16]. Particularly, MERS became endemic in Saudi Arabia and other Middle Eastern countries [17, 18]. At this point, we cannot exclude the possibility of other coronavirus outbreaks in the future. The following is a review of the current status of epidemiology, diagnosis, therapeutics, and vaccines against COVID-19 and related coronaviruses.

## Epidemiology

**Fig. 1:**
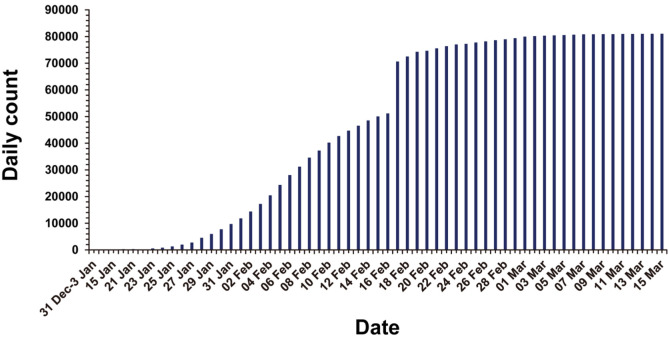
Cumulative confirmed cases of COVID-19 in China, as of 15 March, 2020. Sudden increase of the cases in China on February 17 is due to the change in COVID-19 diagnostic criteria.

**Fig. 2:**
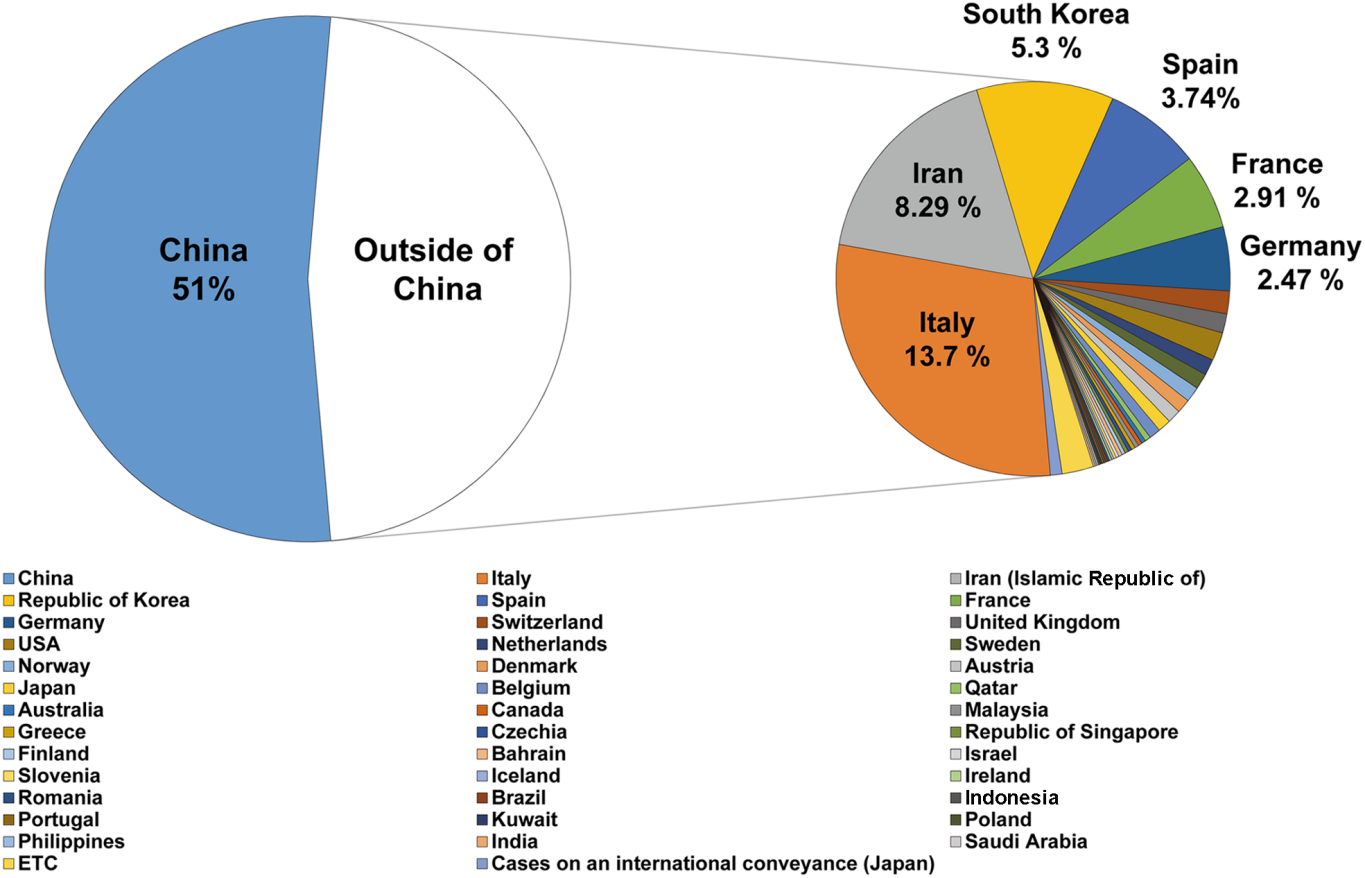
Global distribution of COVID-19 confirmed cases, as of 15 March, 2020. Distribution of the confirmed cases of COVID-19 in each country is presented in the diagram.

The number of COVID-19 cases reported to the WHO has been growing since the first report of COVID-19 in December 2019 from the WHO China Country Office [19]. The infection began to spread from the Huanan seafood wholesale market in Wuhan, China, while the exact infection route of the first case remains unclear. The number of confirmed cases in China grew until mid-February 2020. Then, the number of daily new cases in China started to decrease from late-February 2020 (Fig. 1). A sudden increase of the cases in China on February 17 is due to the change in COVID-19 diagnostic criteria. At the time of writing (March 19, 2020), COVID-19 cases continue to be reported globally from over 170 countries. As of March 15, 2020, 153,517 laboratory-confirmed COVID-19 cases with 5,735 deaths (approximately 3.8% mortality) have been reported according to WHO [20, 21] (Fig. 2).

**Fig. 3:**
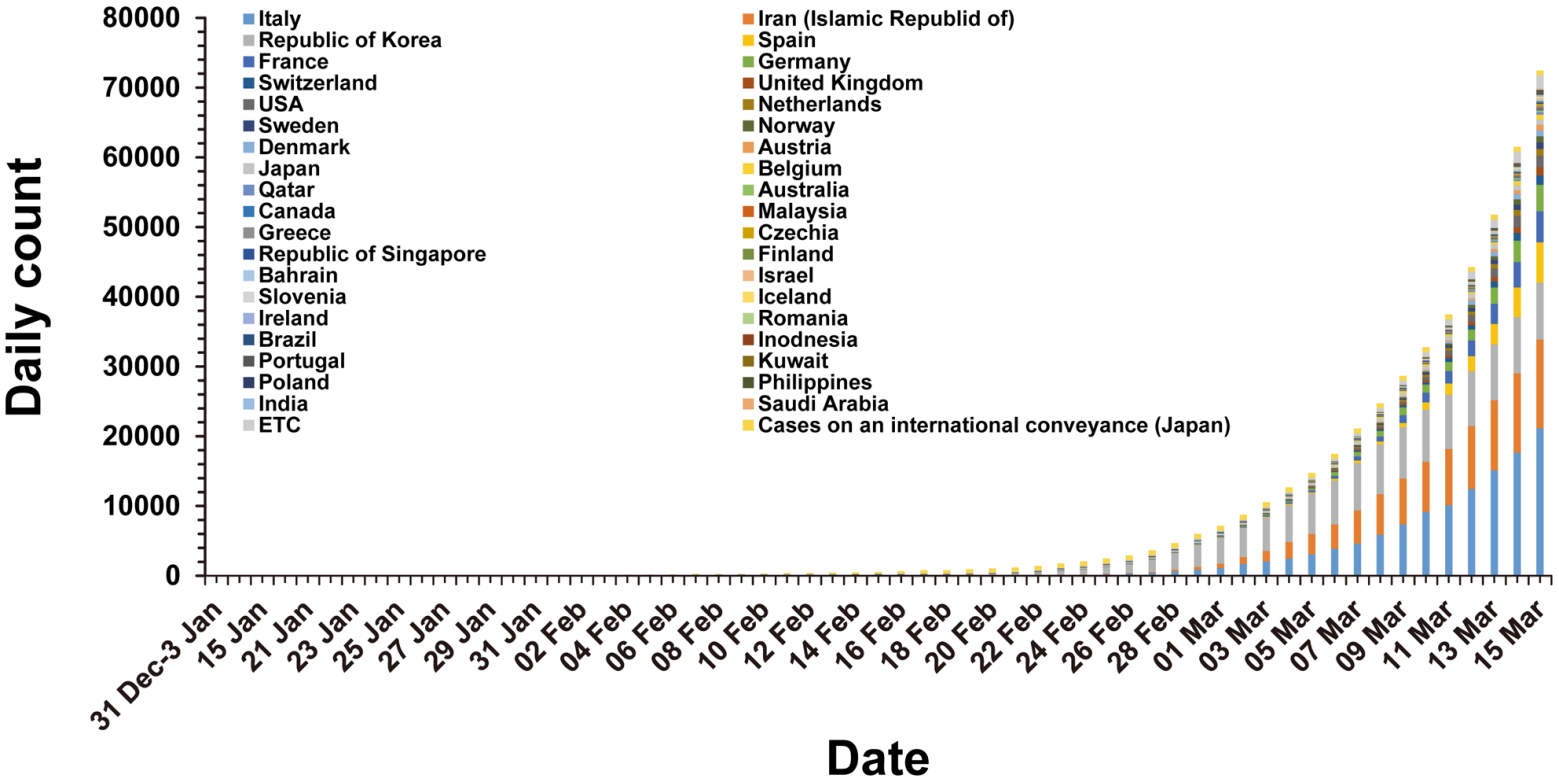
Cumulative confirmed cases of COVID-19 outside of China, as of 15 March, 2020.

**Fig. 4:**
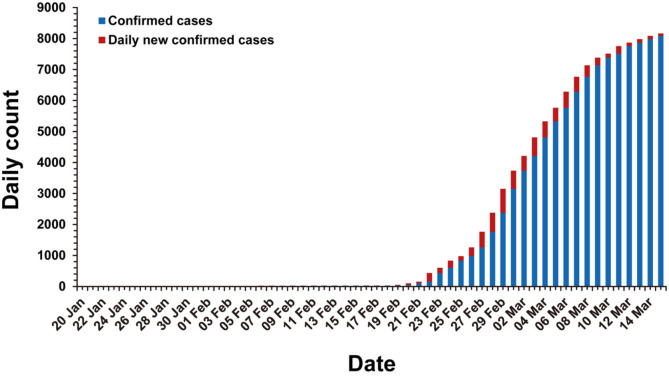
Cumulative confirmed cases of COVID-19 in South Korea, as of 15 March, 2020. Blue bar represents the cumulative confirmed cases before the indicated date. Red bar represents the newly confirmed cases at the indicated date.

In the early stages of the global COVID-19 spread, the cases identified outside of China were mostly travelers who were infected in China and then traveled to areas outside of China. Countries outside of China that reported travel-associated COVID-19 cases were Singapore, Japan, Republic of Korea, Malaysia, Vietnam, Australia, United States of America, Germany, etc. [22]. Unfortunately, COVID-19 has begun to spread domestically in South Korea, Italy, Iran, and Japan from mid-February 2020 [23](Fig.3). Particularly, in the Republic of Korea, the spread of COVID-19 had been well managed until mid-February. The number of confirmed cases in South Korea was 31 on February 18, 2020 [24] and most of these cases were travelers from China or their close contacts. However, COVID-19 infections among a religious group in the Daegu metropolitan area and a nearby hospital triggered a sudden spread to other major domestic cities in South Korea in mid-February (Fig.4). As a result, a week later, the confirmed cases soared to 763 and 74.6% of those cases were tied to the event (as of February 24, 2020) [23]. On March 1, the total number of confirmed cases reached 3,526, among which 59.5% belonged to the religious group-related cases [25].

The mortality rate of SARS-CoV-2 (3.8%) [20] is lower than that of SARS-CoV (10%) [26] or MERS-CoV (37.1%)[27], but the number of relative infection cases is more than 10 times higher. Accumulating reports revealed that SARS-CoV-2 can be transmitted from people who are asymptomatic or have mild infections [28-30]. These features may explain the sudden epidemic spreading of the virus.

## Diagnosis

To detect this novel coronavirus, molecular-based approaches are the first line of methods to confirm suspected cases. Nucleic acid testing is the main technique for laboratory diagnosis. Other methods such as virus antigen or serological antibody testing are also valuable assays with a short turnaround time for the detection of novel coronavirus infection [31, 32]. As with other emerging viruses, the development of methods to detect antibodies and viral antigens are started after the identification of the viral genome.

**Fig. 5:**
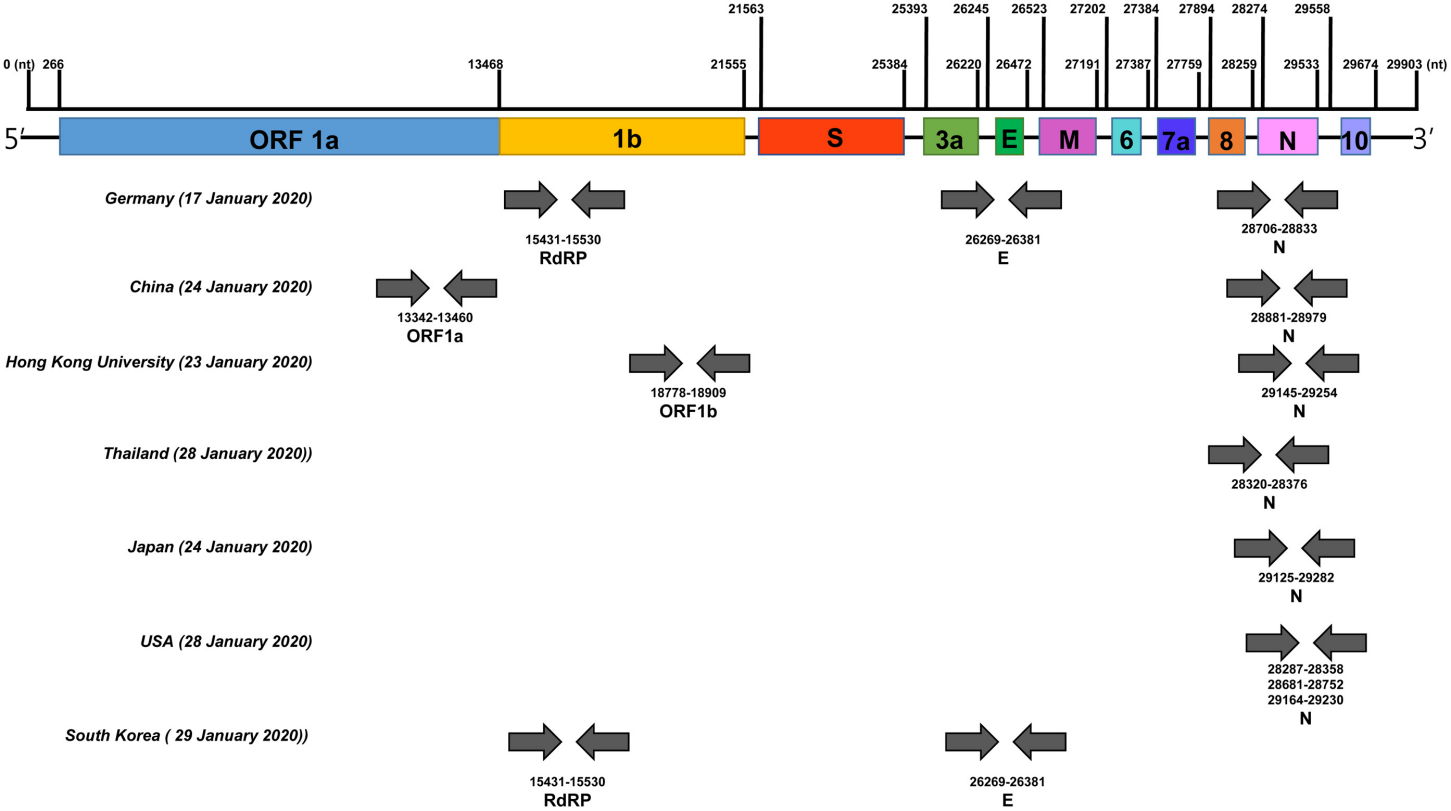
A representative diagram showing currently available diagnostic primer sets on SARS-CoV-2 genome. Numbers represent genome positions according to SARS-CoV-2 isolate Wuhan-Hu-1 (GenBank: MN908947.3). Each primer set for the diagnosis was indicated by grey arrows.

The genomic sequence of SARS-CoV-2 was released immediately to public databases after the start of the outbreak in Wuhan, China on January 10, 2020 (Wuhan-Hu-1, GenBank Accession No. MN908947) [33]. The World Health Organization (WHO) currently recommends that all patient samples with suspected SARS-CoV-2 should be isolated from respiratory tract specimens (including nasal and pharyngeal swabs, sputum, or bronchoalveolar lavage fluid) then shipped to authoritative laboratories for nucleic acid amplification diagnostic testing. During international health emergencies, the real-time RT-PCR assay has shown to be a sensitive and specific method to detect respiratory pathogens in patients with an acute respiratory infection [34]. The presence of SARS-CoV-2 in respiratory specimens was detected by real-time RT-PCR and next-generation sequencing. For the rapid development of real-time RT-PCR diagnostic tests, the genome sequence was used to design specific primers and probes to detect the SARS-CoV-2 [35]. All assays can use viral RNA extracted from SARS-CoV as positive control. The primers and probes targeting specific genes of SARS-CoV-2 were used in real-time RT-PCR assays as diagnostic tests (Fig. 5). The first open reading frames (ORF 1a and 1b), RNA-dependent RNA polymerase gene (RdRp), envelope (E), and nucleocapsid (N) have become key diagnostic targets for SARS-CoV-2 identification. Some countries hosting institutions have shared their protocols and provided specific sequences of target primers on the WHO public database [36].

In the Republic of Korea, since the first confirmed case of SARS-CoV-2 on January 20, 2020, the Korea Centers for Disease Control and Prevention (KCDC) rapidly applied WHO recommended RT-PCR assays and approved various in vitro diagnostic test kits. The diagnostic test kits have been distributed to public health departments and hospitals to prevent the COVID-19 spread in communities.

## Antiviral Therapies

During the COVID-19 outbreak, some potential antiviral drugs are being urgently administered to patients with COVID-19 (Table 1). Those drugs are described in detail below.

## Nucleoside Analogs

Nucleoside analogs have been used as antiviral agents. Nucleoside analogs generally interfere with cellular nucleotide synthesis pathways and cause termination of viral genome replication by the accumulating mutations and by blocking the entry of incoming natural nucleotides. In a broad spectrum of RNA viruses, nucleoside analogs act as inhibitors of viral RNA synthesis. Nucleoside analogs target the RNA-dependent RNA polymerase that is responsible for the replication of viral RNA [37-39].

Favipiravir (T-705) is a guanine analog approved for treatment against influenza virus infection in Japan and also can effectively inhibit replication of Ebola, yellow fever, chikungunya, norovirus, and enterovirus [40]. A recent study suggested that favipiravir is a potential candidate for treatment of SARS-CoV-2 infection showing effective antiviral activities in Vero E6 cells (EC_50_ = 61.88 μM) [41]. For the treatment of patients with COVID-19, favipiravir was used in combination therapy with other antiviral agents such as interferon-α (ChiCTR2000029600) or baloxavir marboxil (ChiCTR2000029544).

Among the approved nucleotide analogs, ribavirin is also a guanine analogue for treatment against hepatitis C virus (HCV) and respiratory syncytial virus (RSV) infection and has been used to treat patients with SARS or MERS [42-44]. However, the drug showed side effects such as anemia in high dose treatment. The efficacy and safety of the drug are uncertain [43, 44]. For COVID-19 treatment, ribavirin was used in combination therapy with pegylated interferon (ChiCTR2000029387) to stimulate innate antiviral responses, which was given in much lower doses to minimize side effects. The combination therapies with interferon should be monitored carefully.

Remdesivir (GS-5734) is an adenine analog that has a similar chemical structure compared with tenofovir alafenamide, an approved HIV reverse transcriptase inhibitor [45]. Remdesivir also showed antiviral activity against MERS-CoV (IC50 = 0.074 μM) and SARS-CoV (IC50 = 0.069 μM) in human airway epithelial (HAE) cell, and, can inhibit MERS-CoV replication in mice [46]. This drug has been developed for the treatment of Ebola virus infection and the therapeutic efficacy was confirmed in a phase III clinical trial [47]. In the USA, the first SARS-CoV-2-infected patient was reported and administered remdesivir [48]. For the inhibition of SARS-CoV-2, the antiviral activity of remdesivir was tested in Vero cells (EC_50_ = 0.77 μM) [41]. Remdesivir has emerged as the most promising candidate for the treatment of SARS-CoV-2 infection. Recently, two phase III clinical trials were initiated to test remdesivir in patients with SARS-CoV-2 (NCT04252664 and NCT04257656). The therapeutic efficacy and safety of favipiravir and remdesivir still need to be confirmed by clinical research in patients with SARS-CoV-2.

## Chloroquine

An approved small-molecule agent, chloroquine is a cheap and safe drug for the treatment of malaria and sequesters protons into lysosomes to increase the intracellular pH. Chloroquine also has potential broad-spectrum antiviral activities by inhibiting endosomal acidification, which is required for virus-host cell fusion [49, 50]. Previous studies determined the antiviral activity of chloroquine against SARS, MERS, HIV, Ebola, Hendra, and Nipah viruses in vitro [51-54]. Chloroquine also showed inhibitory effects against SARS by interfering with glycosylation of the cellular receptor of SARS [52]. A recent in vitro study revealed that chloroquine is a promising candidate antiviral agent against SARS-CoV-2 infection in Vero E6 cells (EC_50_ =1.13 μM) [41]. To meet urgent clinical demand, chloroquine is now being evaluated in clinical trials (ChiCTR2000029609). For the treatment of COVID-19 cases in China, patients were treated with chloroquine to test the efficacy and safety of this antiviral agent candidate against SARS-CoV-2 infection. The results of trials demonstrated that chloroquine inhibits the exacerbation of COVID-19 [55]. Based on this finding, the experts from the government and organizer of clinical trials suggested that chloroquine is a promising antiviral agent against SARS-CoV-2.

## Protease Inhibitors

Protease inhibitors are attractive candidates for antiviral drugs. The viral genome encodes polyproteins which are cleaved by viral proteases for viral gene expression and replication. Protease inhibitors prevent viral gene replication by binding to enzymes that are responsible for proteolytic cleavage [56, 57].

Both lopinavir and ritonavir are protease inhibitors, approved as HIV drugs, and have been reported to have antiviral activities against SARS and MERS [58, 59]. For treatment of SARS-CoV-2, clinical trials (ChiCTR2000029539) have been initiated to test the antiviral activity of HIV protease inhibitors in patients. However, the antiviral efficacy of HIV protease inhibitors in coronavirus proteases is debatable.

There is no specific antiviral agent against emerging novel coronavirus even though great efforts to develop effective antiviral drugs have been made by targeting of virus protease, polymerase, and host proteins following the MERS and SARS epidemics.

In the Republic of Korea after initial reports of an emerging novel coronavirus, experts from the government and academy initiated projects for the screening of potential antiviral drugs against SARS-CoV-2. These projects are supported by the Ministry of Science and ICT (MSIT) of Republic of Korea [60]. To identify novel treatments for patients infected with COVID-19, repurposing of FDA-approved drugs is also a realistic approach.

## Current Status of COVID-19 Vaccine Development

Vaccines are the most effective strategy for preventing infectious disease since they are more cost-effective than treatment, and reduce morbidity and mortality without long-lasting effects [61, 62]. Preventive and therapeutic vaccines will be of fundamental value as the most obvious way to protect global health [62, 63].

Over the past two decades, three human coronaviruses (SARS-CoV, MERS-CoV, and SARS-CoV-2) emerged worldwide, causing considerable threat to global health [64]. However, there is still no approved vaccines for human coronaviruses. Research groups around the world are accelerating the development of COVID-19 vaccines using various approaches. Precise recognition mechanisms between the virus surface proteins and the host receptors are important for understanding of cross-species transmission and host tropism and for the establishment of animal models for vaccine development [65]. The spike (S) protein of coronaviruses is an important target for vaccine development because it mediates the infection mechanism through receptor binding of host cells [65-67]. The S protein of human infectious coronaviruses recognizes various host receptors, including ACE2, APN, and DPP4 (Table 2). SARS-CoV-2 and SARS-CoV use ACE2 as a receptor. The homology between SARS-CoV-2 and SARS-CoV is approximately 75% for the spike (S) protein-RBD [68]. The S1 subunit of S protein contains a receptor-binding domain (RBD) and the S2 subunit is necessary for membrane fusion between host cells and viruses [12, 69]. The following describes the current status of vaccine development against COVID-19 through various approaches.

### Recombinant Subunit Vaccine

In general, subunit vaccines are advantageous over other types of vaccines in that they are highly safe and have fewer side effects by inducing the immune system without introducing infectious viruses [12]. Subunit-based vaccine development studies have also reported enhancement of T cell immune responses and generation of high titer neutralizing antibodies in vivo [70, 71].

Clover Biopharmaceuticals is pre-clinical testing a recombinant subunit vaccine based on the trimeric S protein (S-Trimer) of the SARS-CoV-2 [72]. The S protein contains three S1 heads and a trimeric S2 stalk [67]. Clover Biopharmaceuticals confirmed the generation of a native-like trimeric viral spike in mammalian cell culture-based expression system and the detection of antigen-specific neutralizing antibodies in the sera of fully-recovered COVID-19 patients [72]. Recently, Clover Biopharmaceuticals and GSK announced a partnership to improve immune response by introducing GSK’s adjuvant system to S-Trimer [73].

The University of Queensland is developing subunit vaccines using the “molecular clamp,” a transformative technology [74]. A molecular clamp is a polypeptide that stabilizes a surface protein and improves recognition of the correct antigen, thereby inducing stronger immune responses. This vaccine platform can be readily applicable to a wide range of enveloped viruses and their product can be rapidly manufactured [74]. The University of Queensland has applied GSK’s adjuvant system for the development of an effective vaccine and entered partnership with CEPI [73, 75].

### DNA Vaccine

DNA vaccines represent an innovative approach by direct injection of plasmids encoding the antigens, accompanied with a wide range of immune responses [76]. These advantages are applied with prophylactic vaccines and therapeutic vaccines. Recently, various DNA vaccine platforms have been developed to improve the efficacy of vaccines by using electroporation to deliver plasmids and adding adjuvant to enhance the immune responses [77].

Inovio Pharmaceuticals, in collaboration with Beijing Advaccine Biotechnology, has started pre-clinical trials for DNA vaccine (INO-4800) against COVID-19 [78]. INO-4800 induces activation of T cells by delivering DNA plasmids that express the SARS-CoV-2 spike [79]. This vaccine platform has advantages that can produce therapeutic antibodies and activate immune cells by delivering the vaccines intradermally into the patient. Inovio Pharmaceuticals is preparing for phase I trials in the U.S.A. and China with support from the Coalition for Epidemic Preparedness Innovations (CEPI) [80].

### mRNA Vaccine

mRNA vaccines are rapidly developing technologies to treat infectious diseases and cancers. mRNA-based vaccines contain mRNAs encoding the antigens, which are translated at the host cellular machinery by vaccination [61, 81]. mRNA vaccines have advantages over conventional vaccines, by the absence of genome integration, the improved immune responses, the rapid development, and the production of multimeric antigens [81].

Moderna, Inc. has started phase I clinical trials for mRNA-1273, an mRNA vaccine, encoding viral spike (S) protein of SARS-CoV-2. It was designed in collaboration with the National Institute of Allergy and Infectious Diseases (NIAID) [82]. In contrast to conventional vaccines which are produced in a cell culture system, Moderna’s mRNA vaccine is designed in silico, which enables the rapid development and evaluation of vaccine efficacy [83]. Moderna Inc. is preparing a phase I study with financial support from CEPI [75].

### Other Vaccine Approaches

Genexine Inc. is developing a COVID-19 vaccine using Hyleukin-7 platform technology [84]. Hyleukin-7 platform enhances the immune responses by fusion of interlekin-7 (IL-7) to hyFc, designed to hybridize IgD and IgG4 for long-acting effects of Fc fusion proteins [84, 85]. IgD has a flexible hinge structure that maximizes biological activity of Fc-fusion protein. IgG4 has an unexposed junction site that minimizes adverse immunogenicity by preventing antibody-dependent cellular cytotoxicity (ADCC) and complement-dependent cytotoxicity (CDC) [86, 87]. Genexine Inc. has reported the improved vaccine efficacy showing the accumulation of pulmonary T cells and the increase of plasmacytoid dendritic cells by treatment of Fc-fused IL-7 in influenza A virus infection model [88].

Together with the vaccine technologies described above, some known hurdles also must be overcome for successful vaccine development. Antibody-dependent enhancement (ADE) of disease is a phenomenon in which virus-specific antibodies facilitate virus entry into the host cell via the Fc-receptor pathway, leading to enhanced virus infection [89]. In other words, the antibodies generated by vaccination can promote viral infectivity. ADE effects have been reported in some vaccines against Dengue and Zika viruses [90]. Although the occurrence of ADE of SARS-CoV-2 has not yet been clearly demonstrated, potential ADE of MERS-CoV and SARS-CoV was observed in an in vitro model system [91, 92]. Various approaches have been introduced in vaccine development to avoid adverse effects by ADE. For example, nucleocapsid (N) protein maybe an alternative target to overcome ADE. Since N protein is not a virus surface protein, antibodies induced by N protein-based vaccine will not be able to facilitate virus entry. DNA vaccine candidates targeting SARS-CoV N protein as an immunogen can generate N-specific humoral and cellular immune response [93]. Another major hurdle for vaccine development is higher mutation rates of RNA viruses compared to DNA viruses, resulting in higher genetic diversity [94]. Moreover, the RBD of the S protein is the most variable region in the coronavirus genome [95]. Therefore, the ADE and the higher genetic diversity should be considered as important factors for vaccine design and antibody-based drug development against SARS-CoV-2.

## Summary

Despite tremendous global efforts to contain SARS-CoV-2, the virus’s spread has reached a pandemic level. Although the development of therapeutics and vaccines for the treatment of COVID-19 is still in its early stage, there has been some significant progress in the research area from complete genome sequencing of SARS-CoV-2 to the beginning of clinical trials with COVID-19 vaccines. Continued international efforts are required to solve the remaining unanswered questions about the novel coronavirus, including the animal sources of infection, transmissibility, and virulence of the virus.

Rapid complete genome analysis of SARS-CoV-2 and international sharing of the information have so far allowed us to produce faster and more accurate diagnostic tools. Currently, most of the developed diagnostic tools are qRT-PCR-based diagnostic tools, which require longer sample preparation and analysis time, delaying necessary actions for COVID-19 patients in the field. As COVID-19 spreads continuously all over the world, the development of rapid diagnostic methods, which can be tested in the field, is urgently required.

It has been reported that some drugs are known to be effective for the treatment of COVID-19 patients. However, the lack of clinical evidence may lead to unpredictable clinical prognosis. At the moment, quick screening of therapeutic agents for the repurposing of FDA-approved and well-characterized drugs may be a more practical approach in epidemics. Considering the seriousness of the recent outbreaks of zoonotic coronaviruses, therapeutic agents for pan-coronaviruses should be developed to cope with future outbreaks.

Together with the development of diagnostics and therapeutics, various pharmaceutical companies and institutions have announced the beginning of vaccine development as well. However, enormous funds and long-term studies are required for clinical research for the development of vaccines against infectious diseases. Despite the serious public health threat caused by viral outbreaks, the commercial markets for vaccines, particularly against emerging infectious diseases, are quite limited due to the high cost and time required for vaccine development and the uncertainty of profitability. This development environment of vaccines has created a huge gap between academic research and commercial markets. Thus, well-organized, long-term research programs should be established with internationally supported projects for the development of vaccines against emerging infectious diseases. For this reason, CEPI (Coalition for Epidemic Preparedness Innovations) was launched at Davos in 2017 to link academics and pharmaceutical companies and to provide financial support for the development of vaccines against emerging infectious disease [75]. Currently, a global vaccine research development pipeline for infectious diseases has been established and its clinical trials have been funded by CEPI. In addition to these global movements, the South Korean government and the Korea Centers for Disease Control and Prevention (KCDC) have announced urgent research projects for the development of vaccines and drugs against COVID-19.

Outbreaks caused by zoonotic coronaviruses can result not only in severe human casualties but also in global economies disrupting manufacturing supply chains and decreasing market demand. Therefore, cooperation of various institutions, academics, governments, and pharma-ceutical companies is inevitably necessary to prevent further spreading of the current COVID-19 and the future outbreaks.

## Figures and Tables

**Table 1:** List of candidate therapeutic agents for SARS-CoV-2 treatment.

Candidate therapeutic	Market name	Manufacturer	Target disease	EC_50_(COVID-19)	Status of clinical trials
Favipiravir (T-705)	Avigan	Toyama	Influenza	61.88 μM [41]	Under clinical trial for COVID-19 (ChiCTR2000029548)
		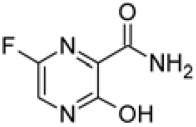			
Remdesivir (GS-5734)	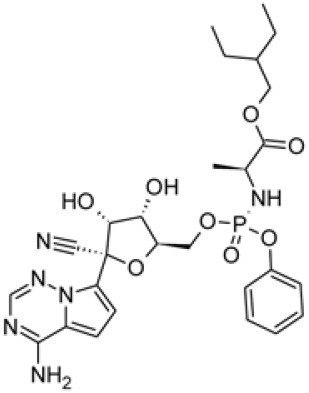	Gilead	Ebola	0.77 μM [41]	Phase II clinical trial for Ebola (NCT03719586); Under phase III clinical trials for COVID-19 (NCT04252664)
Chloroquine/Hydroxychloroquine	Aralen/Plaquenil	Sanofi	Malaria	1.13 μM [41]	Under clinical trials for COVID-19 (^a^)
		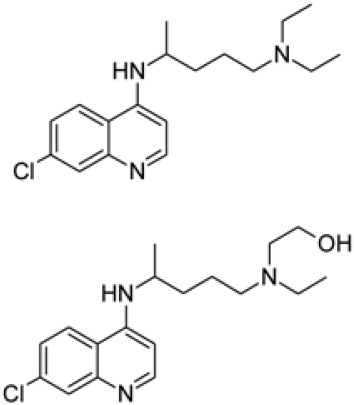			
Lopinavir and Ritonavir	Kaletra	Abbott	HIV	−^b^	Under clinical trials for SARS [59] Under clinical trial for COVID-19 (ChiCTR2000029539)
		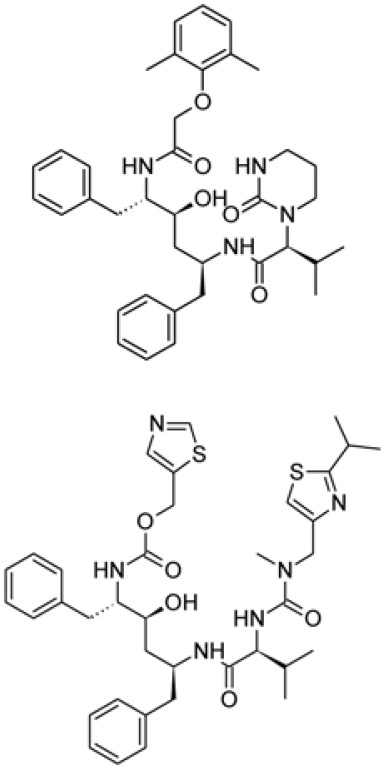			
Pegylated interferon with ribavirin	Virazole	Valeant	HBV, HCV	109 μM [41]	Under clinical trial for COVID-19 (ChiCTR2000029387)
		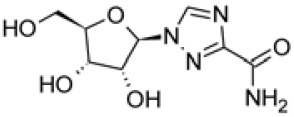			

^a^ChiCTR2000029939, ChiCTR2000029935, ChiCTR2000029899, ChiCTR2000029898, ChiCTR2000029868, ChiCTR2000029837, ChiCTR2000029826, ChiCTR2000029803, ChiCTR2000029762, ChiCTR2000029761, ChiCTR2000029760, ChiCTR2000029740, ChiCTR2000029609, ChiCTR2000029559, and ChiCTR2000029542

^b^Undetermined

**Table 2:** Host tropisms and receptors of coronaviruses.

Virus	Host	Cellular receptor	Reference
Alphacoronavirus			
HCoV-229E	Human	APN^a^	[96]
HCoV-NL63	Human	ACE2^b^	[97]
FCoV	Feline	APN	[98]
CCoV	Canine	APN	[98]
TGEV	Porcine	APN	[99]
Betacoronavirus			
SARS-CoV	Human	ACE2	[100]
MERS-CoV	Human	DPP4^c^	[101]
HCoV-OC43	Human	9-O-acetylated sialic acid	[102]
HCoV-HKU1	Human	9-O-acetylated sialic acid	[103]
MHV	Mouse	CEACAM^d^	[104]

^a^Aminopeptidase N

^b^Angiotensin-converting enzyme 2 (ACE2)

^c^Dipeptidyl peptidase 4 (DPP4/CD26)

^d^Carcinoembryonic antigen-related cell adhesion molecules (CEACAM)
